# Activating Transcription Factor 4 Confers a Multidrug Resistance Phenotype to Gastric Cancer Cells through Transactivation of SIRT1 Expression

**DOI:** 10.1371/journal.pone.0031431

**Published:** 2012-02-17

**Authors:** Hongwu Zhu, Limin Xia, Yongguo Zhang, Honghong Wang, Wenjing Xu, Hao Hu, Jing Wang, Jing Xin, Yi Gang, Sumei Sha, Bin Xu, Daiming Fan, Yongzhan Nie, Kaichun Wu

**Affiliations:** Department of Gastroenterology and State Key Laboratory of Cancer Biology, Xijing Hospital, Fourth Military Medical University, Xi'an, People's Republic of China; Vanderbilt University Medical Center, United States of America

## Abstract

**Background:**

Multidrug resistance (MDR) in gastric cancer remains a major challenge to clinical treatment. Activating transcription factor 4 (ATF4) is a stress response gene involved in homeostasis and cellular protection. However, the expression and function of ATF4 in gastric cancer MDR remains unknown. In this study, we investigate whether ATF4 play a role in gastric cancer MDR and its potential mechanisms.

**Methodology/Principal Findings:**

We demonstrated that ATF4 overexpression confered the MDR phenotype to gastric cancer cells, while knockdown of ATF4 in the MDR variants induced re-sensitization. In this study we also showed that the NAD^+^-dependent histone deacetylase SIRT1 was required for ATF4-induced MDR effect in gastric cancer cells. We demonstrated that ATF4 facilitated MDR in gastric cancer cells through direct binding to the SIRT1 promoter, resulting in SIRT1 up-regulation. Significantly, inhibition of *SIRT1* by small interfering RNA (siRNA) or a specific inhibitor (EX-527) reintroduced therapeutic sensitivity. Also, an increased Bcl-2/Bax ratio and MDR1 expression level were found in ATF4-overexpressing cells.

**Conclusions/Significance:**

We showed that ATF4 had a key role in the regulation of MDR in gastric cancer cells in response to chemotherapy and these findings suggest that targeting ATF4 could relieve therapeutic resistance in gastric cancer.

## Introduction

Multidrug resistance is usually the main cause for failure of chemotherapy against malignant tumors, including gastric cancer [1].The term multidrug resistance is classically used to define a resistance phenotype where cells become resistant simultaneously to different drugs with no obvious structural resemblance and with different cellular targets [2]. MDR occurs more frequently with novel drugs that have more significant effectiveness after their first application in cancer treatment. The clinical usefulness of multiple drugs is limited by both natural and acquired tumor cell resistance, which almost always is multifactorial in nature [3]. The factors that may affect drug sensitivity include: accelerated drug efflux, drug activation and inactivation, alterations in the drug target, DNA methylation, processing of drug-induced damage, and evasion of apoptosis [4].

Gastric cancer is relatively insensitive to chemotherapeutics. The MDR mechanisms in gastric cancer cells have been broadly investigated in our laboratory and elsewhere [1], [4], [5], yet they have not been fully elucidated, indicating that other unknown molecules or pathways may be involved in the development of MDR.

In mammalian cells, eukaryotic translation initiation factor 2 α subunit (eIF2α) is phosphorylated by different eIF2α kinases in response to different stress signals, including anoxia/hypoxia, endoplasmic reticulum stress, amino acid deprivation, and oxidative stress. This phosphorylation event leads to a rapid decrease in global protein biosynthesis concurrent with induction of translational expression of genes, including *ATF4* that function to alleviate cellular damage from stress [6], [7]. Although ATF4 may play a pro-apoptotic role under conditions of severe or prolonged stress, *ATF4* is a potent stress-responsive gene thought to play a protective role by regulating cellular adaptation to adverse circumstances in the integrated stress response (ISR) [8], [9], [10]. Recently, overexpression of ATF4 was reported to be prominent in a wide variety of tumors and to protect tumor cells against multiple stresses, as well as a range of cancer therapeutic agents [11], [12], [13], [14], [15], [16], [17]. The potential mechanisms responsible for this protection include autophagy induction, promotion of DNA damage repair, and up-regulation of intracellular glutathione [12], [13], [14], [17]. However, the expression and function of ATF4 in gastric cancer MDR remains unknown.

In this study, we reported that ATF4 was significantly up-regulated in the MDR response of gastric cancer cells compared with parental control cells. Knockdown of *ATF4* by siRNA significantly sensitized cells with MDR to a variety of chemotherapeutic agents, whereas up-regulation of ATF4 in SGC7901 and AGS cells rendered them multidrug resistant. We also showed that ATF4 promoted gastric cancer MDR partly through up-regulating expression of SIRT1. And SIRT1 inhibition could partly reverse the gastric cancer MDR phenotype mediated by ATF4. These data suggest that targeting ATF4 may provide a novel therapeutic option for reversing clinical gastric cancer MDR.

## Results

### ATF4 modulates the MDR phenotype of gastric cancer cells

**Figure 1:**
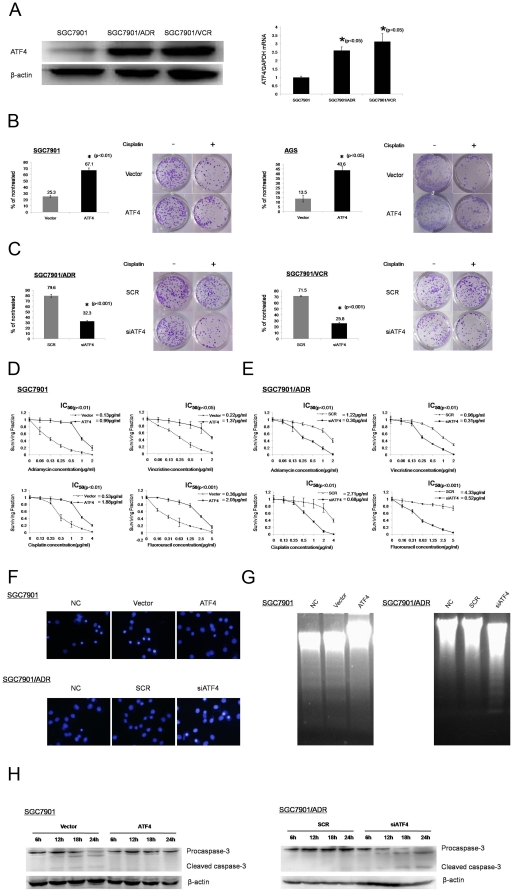
ATF4 modulate the MDR phenotype of gastric cancer cells. (A) The protein and mRNA levels of ATF4 in MDR gastric cancer cells (SGC7901/ADR and SGC7901/VCR) and parental SGC7901 cells were examined by Western blotting and qPCR. β-actin and GAPDH were used as internal control, respectively. Data represent the means ± S.D. of three independent experiments. (B) The response of LV-Vector and LV-ATF4 stably transfected SGC7901 and AGS to cisplatin was tested by colony formation assay. Cell lines were treated continuously with either 0 or 0.25 µg/ml cisplatin for 14 d; media was changed every 3 d. Cells were plated in triplicate, and the experiment was repeated three times. Representative wells are shown. Graphs provide average quantification as a percentage of the nontreated cells. (C) LV-SCR and LV-siATF4 stably transfected SGC7901/ADR and SGC7901/VCR cells were treated continuously with either 0 or 0.5 µg/ml cisplatin for 14 d; media was changed every 3 d. (D) and (E) LV-Vector and LV-ATF4 stably transfected SGC7901 cell lines and LV-SCR and LV-siATF4 stably transfected SGC7901/ADR cells were treated with indicated doses of different drugs for 72 h. *In vitro* drug sensitivity was tested by MTT assay. Data represent the means ± S.D. of three independent experiments. (F) and (G) LV-Vector and LV-ATF4 stably transfected SGC7901 cell lines, LV-SCR and LV-siATF4 stably transfected SGC7901/ADR cells and their respective nontreated counterparts(NC) were grown in fresh medium in the presence of cisplatin at the indicated concentrations (for SGC7901-NC, SGC7901-Vector, and SGC7901-ATF4, 5 µg/ml; for SGC7901/ADR-NC, SGC7901/ADR-SCR, and SGC7901/ADR-siATF4, 10 µg/ml) for 36 h. Then Hoechst 33258 nuclear staining and DNA fragmentation assay were performed. (H) SGC7901 and SGC7901/ADR stable transfected cell lines as above were incubated for additional 6–24 h in fresh medium with indicated concentrations of cisplatin (for SGC7901-Vector and SGC7901-ATF4, 10 µg/ml; for SGC7901/ADR-SCR and SGC7901/ADR-siATF4, 20 µg/ml). At the time indicated, protein extracts were collected and subjected to immunoblot analysis for caspase-3 (uncleaved and cleaved forms). β-actin was used as an internal control.

To determine whether ATF4 is involved in the development of MDR in gastric cancer cells, ATF4 levels were detected by Western blot and qPCR in the SGC7901 cell line and its MDR variants, SGC7901/VCR and SGC7901/ADR. Both protein and mRNA levels of ATF4 were much higher in the resistant cell lines than in parental cells (Fig. 1A).

To investigate whether ATF4 overexpression is sufficient to induce a MDR phenotype in gastric cancer cells, *ATF4* expression cDNA was stably transfected into SGC7901 and AGS cells. First, CDDP sensitivity was tested using a colony formation assay. As shown by the quantification of the colony formation assay, ATF4 overexpression resulted in a nearly 3-fold increase in colony numbers compared with empty vector-expressing cells (Fig. 1B). MTT assays also indicated that the IC_50_ values of SGC7901-ATF4 for ADR, VCR, CDDP, and 5-FU were significantly increased as compared to empty vector transfected cells(Fig. 1D).

As ATF4 levels are elevated in MDR gastric cancer cells, we further wanted to determine whether targeting ATF4 could re-sensitize the MDR cell lines. Knockdown of *ATF4* by siRNA in the SGC7901/ADR and SGC7901/VCR cells led to a 2- to 3-fold reduction in cell number when used in combination with CDDP (Fig. 1C). Data in Fig. 1E also suggest that down-regulation of *ATF4* significantly reverses the resistance of SGC7901/ADR cells in response to chemotherapy.

As inhibition of apoptosis is one of important mechanisms of MDR, we also investigated the capacity of the SGC7901/ADR cells transfected with the specific *ATF4* siRNA to undergo CDDP-induced apoptosis by Hoechst staining and DNA fragmentation assays. Treatment of SGC7901-ATF4 and SGC7901/ADR-SCR cells with the indicated concentrations of CDDP for 36 hours did not induce any apoptosis, as assessed by Hoechst nuclear staining (Fig. 1F) and DNA fragmentation assays (Fig. 1G). In contrast, SGC7901-Vector and SGC7901/ADR-siATF4 cells displayed significant apoptosis, with the more frequent appearance of condensed and fragmented nuclei and DNA ladder formation. Moreover, more obvious cleavage of procaspase-3 was observed after treatment with CDDP in SGC7901-Vector and SGC7901/ADR-siATF4 cells as compared to SGC7901-ATF4 and SGC7901/ADR-SCR cells, respectively (Fig. 1H).

Taken together, these results indicate that ATF4 confers a MDR phenotype to gastric cancer cells and that targeting ATF4 provides a method of sensitizing resistant cells to chemical treatments.

### ATF4 up-regulates the expression of SIRT1, MDR1, Bcl-2, and Bax in gastric cancer cells

Previous studies have reported that cells overexpressing SIRT1 displayed decreased sensitivity to chemotherapy by multiple mechanisms [18], [19], [20], [21]. We were curious to determine whether *SIRT1*, which is a stress-related gene critical to MDR development, could be the downstream target of ATF4 responsible for mediating ATF4-induced MDR in gastric cancer cells.

**Figure 2:**
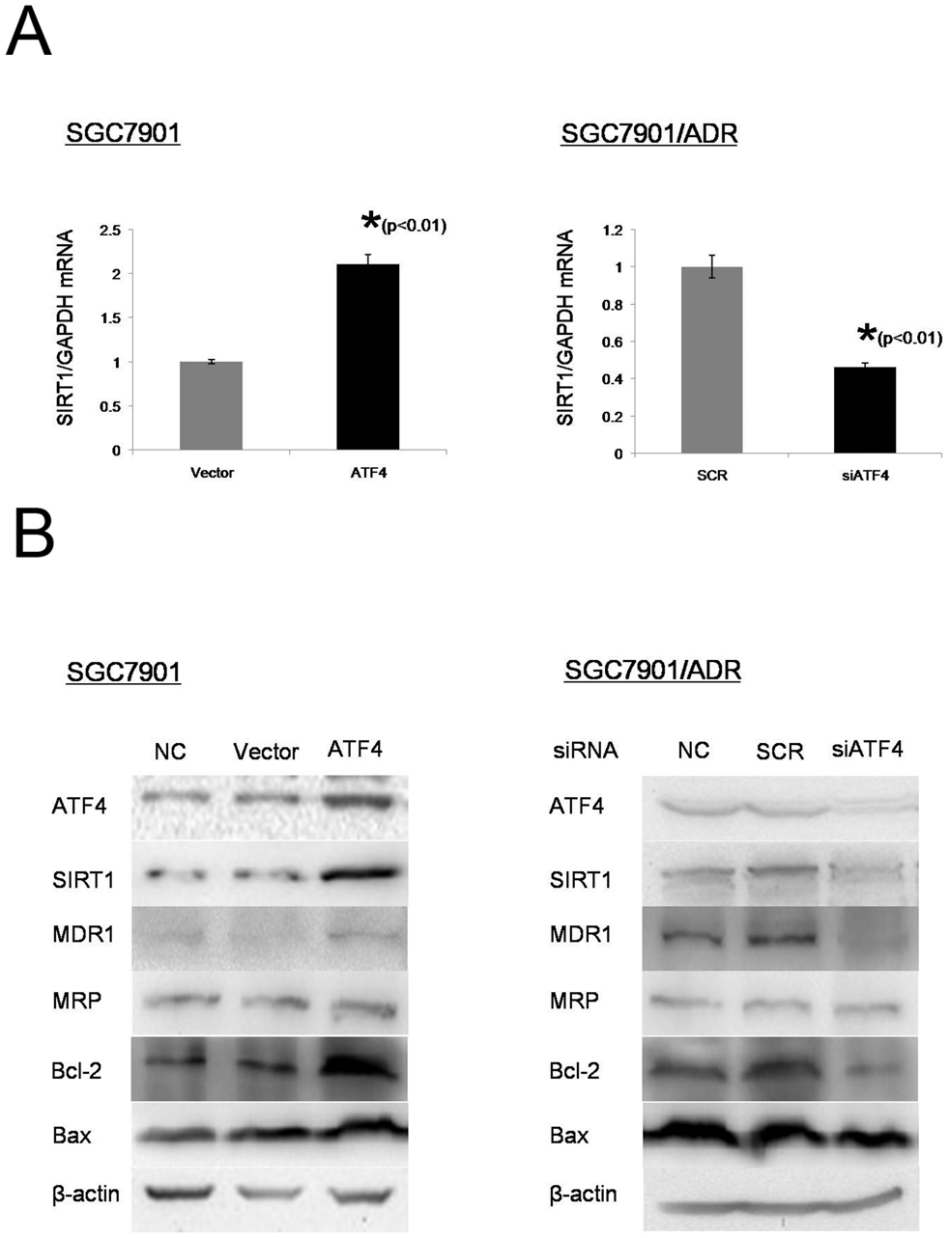
ATF4 up-regulates SIRT1 expression in gastric cancer cells. (A) mRNA levels of SIRT1 in LV-Vector and LV-ATF4 stably transfected SGC7901 cell lines (left) and LV-SCR and LV-siATF4 stably transfected SGC7901/ADR cells (right) were subjected to qPCR. GAPDH were used as an internal control. Data represent the means ± S.D. of three independent experiments. (B) Cell lysates from cells in section A and their respective nontreated counterparts(NC) were blotted with the indicated antibodies. β-actin was used as an internal control.

SIRT1 levels in LV-Vector and LV-ATF4 stably transfected SGC7901 cells were assayed by qPCR and Western blot. The overexpression of ATF4 was associated with increased SIRT1 expression at both the transcriptional (Fig. 2A, left) and translational levels (Fig. 2B, left). In contrast, siRNA knockdown of *ATF4* in SGC7901/ADR cells resulted in a significant reduction of endogenous *SIRT1* expression (Fig. 2A and 2B, right). These results suggest that ATF4 up-regulates *SIRT1* expression in gastric cancer cells.

To further investigate the molecular mechanisms involved in ATF4-related MDR of gastric cancer, we also examined MDR1, MRP, Bcl-2, and Bax expression levels in the gastric cancer cells used above. As shown in Fig. 2B, ATF4-proficient cells expressed more MDR1 as compared to the control cells. Meanwhile, no obvious difference in MRP expression was found in any of these cell lines. Interestingly, both Bcl-2 and Bax expression levels were up-regulated in ATF4-proficient cells, compared with the control cell lines, while the expression of Bax showed only slight changes, indicating that an up-regulation of the Bcl-2 to Bax ratio might suppress the drug-induced apoptosis in ATF4-overexpressing gastric cancer cells.

These results indicate that ATF4 promotes MDR ability of gastric cancer cells through multiple mechanisms.

### ATF4 transactivates SIRT1 promoter activity and directly binds to the SIRT1 promoter

**Figure 3:**
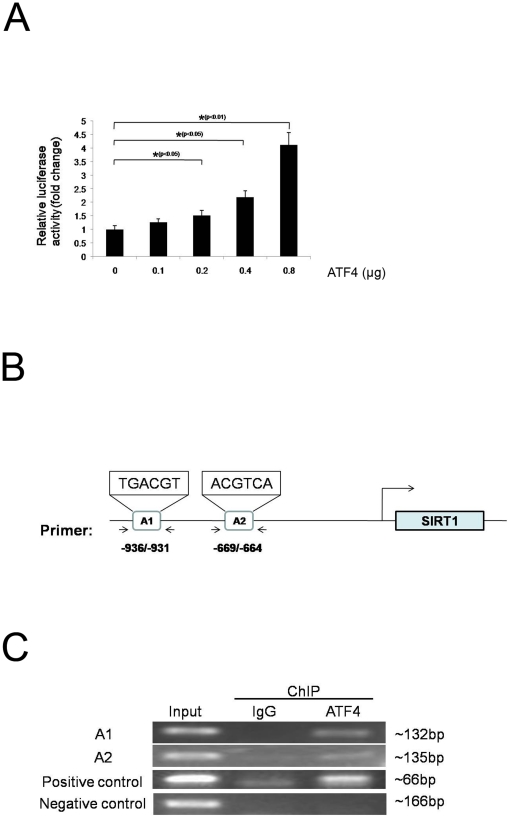
ATF4 transactivates SIRT1 expression through binding to the *SIRT1* promoter. (A) 293T cells were co-transfected with the 1.2 kb *SIRT1* promoter reporter plasmid and the *ATF4* expression plasmid. After 48 hours, luciferase reporter assay was used to detect the *SIRT1* promoter activity. Data represent the means ± S.D. of three independent experiments. (B) A schematic representation of the human SIRT1 gene promoter showing the RT-PCR primers' positions for ChIP analysis. (C) ChIP assay was used to detect the direct binding of ATF4 to the *SIRT1* promoter. SGC7901-ATF4 cells were processed for ChIP using anti-ATF4 antibody. A1 represent the putative distal binding site and A2 represent the putative proximal binding site. The ASNS promoter primers were used as a positive control, and GAPDH primers were used as a negative control.

To determine whether ATF4 mediates *SIRT1* gene transcription, 293T cells were co-transfected with the 1.2 kb *SIRT1* promoter reporter plasmid and the *ATF4* expression plasmid. The luciferase reporter assay showed that the *SIRT1* promoter activity was markedly activated by ATF4 in a dose-dependent manner (Fig. 3A).

In an attempt to gain specific insight into the mechanisms of *SIRT1* induction, we examined the possible induction pathways from ATF4. By analyzing the 5′-flanking sequence of the *SIRT1* gene with bioinformatics softwares (Tfsitescan service, TESS, and Genomatix), two ATF4 putative binding sites were identified within the −950 to −600 bp region of the *SIRT1* promoter (Fig. 3B).

To determine whether *SIRT1* is a direct target of ATF4, ChIP with the ATF4 antibody using SGC7901-ATF4 cells showed enrichment of both binding sites within the *SIRT1* promoter region, indicating that the RNA and subsequent protein level increases of *SIRT1* in ATF4-expressing cell lines are likely due to a direct interaction of ATF4 with the *SIRT1* gene promoter (Fig. 3C).

To investigate the role of the two ATF4 binding sites in regulating SIRT1 transactivation, site-directed mutagenesis was used to mutate these sites. Luciferase reporter assay showed that either mutating the binding site 1 or binding site 2 reduced the SIRT1 promoter activity induced by ATF4. Furthermore, mutation of both binding sites abolished the SIRT1 promoter activity. These results suggested that both ATF4 binding sites are involved in the transactivation of SIRT1 promoter (Fig. S1).

Taken together, these results indicate that SIRT1 is a direct transcriptional target of ATF4.

### *SIRT1* inhibition by siRNA partly reverses the MDR phenotype of ATF4-overexpressing gastric cancer cells

**Figure 4:**
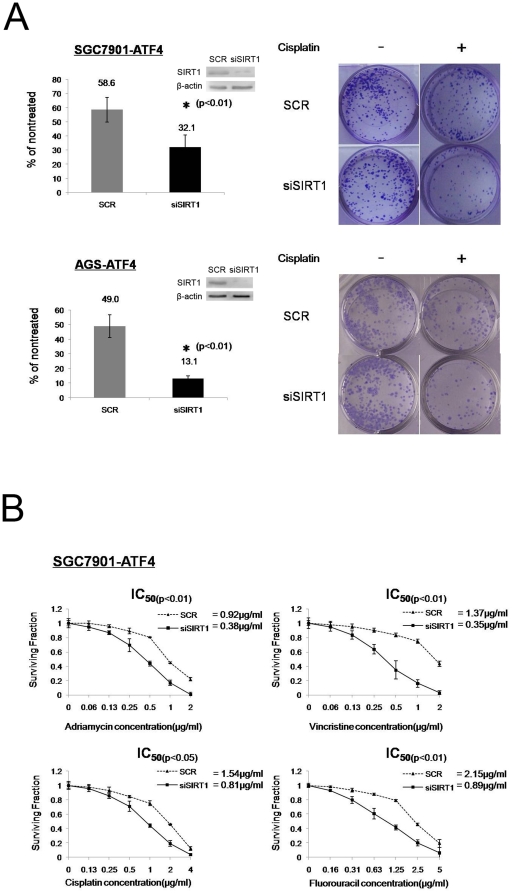
SIRT1 inhibition by siRNA suppressed the ATF4-induced gastric cancer MDR phonotype. (A) SGC7901-ATF4 and AGS-ATF4 cells were transfected with scrambled siRNA (SCR) or SIRT1 siRNA (siSIRT1). Seventy-two hours later, Cell lines were treated continuously with either 0 or 0.25 µg/ml cisplatin for 14 d; media was changed every 3 d. Cells were plated in triplicate, and the experiment was repeated three times. Representative wells are shown. Graphs provide average quantification as a percentage of the nontreated cells. Inset, relative SIRT1 protein expression by Western blot. (B) SGC7901-ATF4 cells were transfected with scrambled siRNA (SCR) or SIRT1 siRNA (siSIRT1). Seventy-two hours later, both cell lines were treated with the indicated doses of different drugs for additional 72 h. *In vitro* drug sensitivity was tested by MTT assay. Data represent the means ± S.D. of three independent experiments.

The identification of ATF4-mediated *SIRT1* expression level increases in gastric cancer cells, prompted us to analyze the role of this pathway in gastric cancer MDR. To address this, we compared the *in vitro* drug sensitivity in ATF4 stably transfected gastric cancer cells after transfection of *SIRT1* siRNA or scrambled siRNA by colony formation and MTT assays. Knockdown of *SIRT1* by siRNA in SGC7901-ATF4 cells led to a >40% reduction in colony number when used in combination with CDDP (Fig. 4A, upper). This effect was also observed in AGS-ATF4 cells (Fig. 4A, lower). Moreover, data from the MTT assay also indicated that knockdown of *SIRT1* could re-sensitize SGC7901-ATF4 cells to chemical drugs, but this did not occur in scrambled siRNA transfected cells (Fig. 4B).

**Figure 5:**
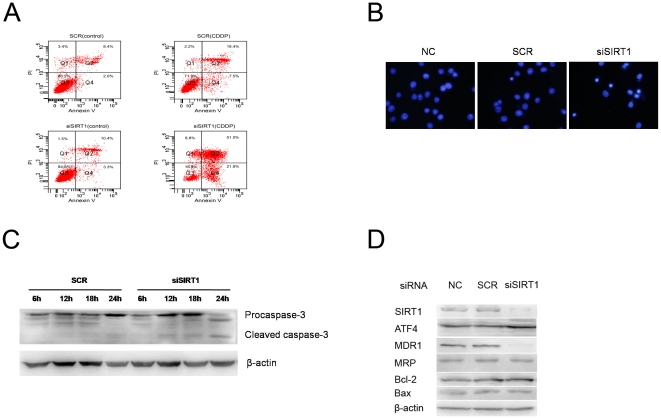
Effect of down-regulation of SIRT1 by siRNA on apoptosis and MDR related molecules. (A) SGC7901-ATF4 cells were transfected with scrambled siRNA (SCR) or SIRT1 siRNA (siSIRT1). Seventy-two hours later, cells were incubated for additional 36 h in fresh medium in the absence or presence of cisplatin at 5 µg/ml. After drug treatment, the cells were labeled with Annexin V and PI. The distribution pattern of live and apoptotic cells was determined by FACS analysis. (B) SGC7901-ATF4 cells were transfected by the same way in section A and then treated with 5 µg/ml of cisplatin for 36 h. Then Hoechst 33258 nuclear staining was performed to detect apoptotic cells. (C) SGC7901-ATF4 cells were transfected by the same way in section A and were incubated for additional 6–24 h in fresh medium with 10 µg/ml of cisplatin. At the time indicated, protein extracts were collected and subjected to immunoblot analysis for caspase-3 (uncleaved and cleaved forms). β-actin was used as an internal control. (D) SGC7901-ATF4 cells were transfected by the same way in section A. Seventy-two hours later, cell lysates were blotted with the indicated antibodies. β-actin was used as an internal control.

To determine whether SIRT1 protects the cells from CDDP-induced apoptosis, SGC7901-ATF4 cells transfected with *SIRT1* siRNA or scrambled siRNA were treated with CDDP and labeled with Annexin V and PI. The apoptotic cells were identified by Annexin V labeling. The apoptotic percentage of SIRT1 siRNA transfected SGC7901-ATF4 cells was significantly higher than that of the control cells (Fig. 5A, 72.8% vs. 25.9%). The appearance of condensed and fragmented nuclei was also increased in SIRT1 siRNA transfected cells compared to the control cells (Fig. 5B). Furthermore, cleavage of procaspase-3 was observed as early as 12 h after treatment with 10 µg/ml CDDP in SIRT1 siRNA transfected SGC7901-ATF4 cells, but not in scrambled siRNA treated cells, even after 24 h of CDDP treatment (Fig. 5C). These results suggest that SIRT1 overexpression suppresses CDDP-induced apoptosis.

To study the effect of down-regulation of SIRT1 by siRNA on MDR associated molecules, we examined MDR1, MRP, Bcl-2, and Bax expression levels in SGC7901-ATF4 cells following transfection with SIRT1 siRNA or scrambled siRNA. Down-regulation of MDR1 was observed in the SIRT1 siRNA-treated cells (Fig. 5D) compared to the control cells. In contrast, no obvious difference of MRP, Bcl-2, and Bax expression levels were found between the samples.

These observations indicate that SIRT1 mediates the ATF4-induced MDR effect in gastric cancer cells.

### Inhibition of SIRT1 activity re-sensitizes ATF4 transfected cells to DNA-damaging agents

**Figure 6:**
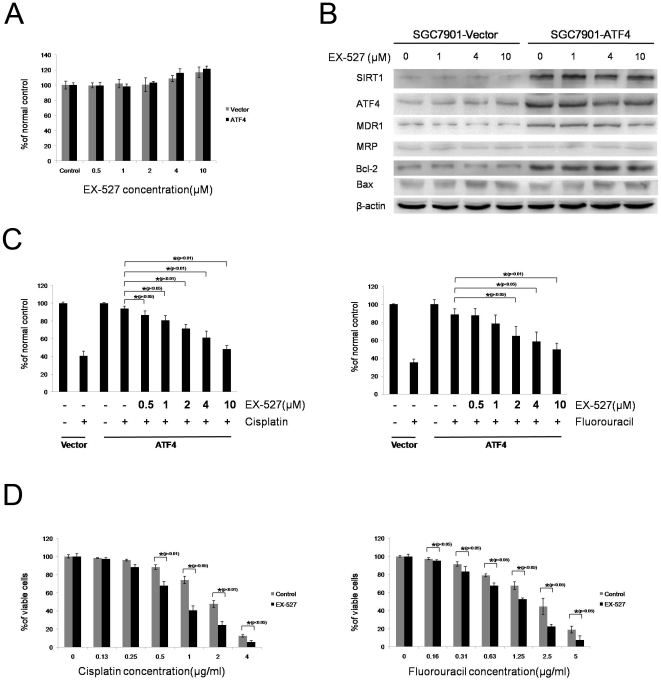
Inhibition of SIRT1 activity reintroduce sensitivity in ATF4-overexpressing cell lines. (A) LV-Vector and LV-ATF4 stably transfected SGC7901 cell lines were incubated with or without the indicated doses of EX-527. Ninety-six hours later, cell viabilities were determined by MTT assay. (B) Stably transfected SGC7901 cell lines in section A were incubated with or without EX-527 (1–10 µM) for 24 h, and total cell lysates were subjected to immunoblotting with the indicated antibodies. β-actin was used as an internal control. (C) SGC7901-ATF4 cells were preincubated with the indicated doses of EX-527 for 24 h. Then SGC7901-Vector and SGC7901-ATF4 cells were exposed to cisplatin (1 µg/ml) or 5-fluorouracil (1.25 µg/ml) for additional 72 h. Cell viabilities were determined by MTT assay. (D) SGC7901-ATF4 cells were preincubated with or without EX-527 (10 µM) for 24 h. Then the cells were exposed to the indicated doses of cisplatin or 5-fluorouracil for additional 72 h. Cell viabilities were determined by MTT assay. All data represent the means ± S.D. of three independent experiments. Graphs provide average quantification as a percentage of the nontreated cells.

To provide evidence that SIRT1 catalytic activity is also responsible for the ATF4-induced MDR, SGC7901-ATF4 cells were pretreated with EX-527, a novel, potent and specific small-molecule inhibitor of SIRT1, and followed by treatment with different chemical drugs. First, we determined the basal cytotoxicity of EX-527 in LV-Vector and LV-ATF4 stably transfected SGC7901 cells. The MTT assay revealed that EX-527 at concentrations up to 10 µM did not inhibit, but rather slightly increased, the viability of both cell lines (Fig. 6A). Next, we examined SIRT1, ATF4, MDR1, MRP, Bcl-2, and Bax expression levels after 24 hours' incubation with or without the indicated doses of EX-527 in the gastric cancer cells used above. Only the expression of MDR1 were down-regulated by EX-527 in a concentration-dependent manner (Fig. 6B). Then we preincubated SGC7901-ATF4 cells with vehicle or EX-527 (0.5, 1, 2, 4, and 10 µM) for 24 h, and then CDDP- and 5-FU-mediated cell death was monitored. As shown in Fig. 6C, EX-527 significantly enhanced the cytotoxicity of both drugs in a dose-dependent manner. We also determined the possible synergistic effect of EX-527 on different doses of CDDP- and 5-FU-mediated inhibition of cell proliferation in SGC7901-ATF4 cells. As expected, 10 µM EX-527 is sufficient to potentiate the cytotoxicity of both drugs (Fig. 6D).

These results suggest that SIRT1 activity also plays a critical role in the ATF4-induced gastric cancer MDR and this role might be mediated partly through MDR1 expression.

## Discussion

MDR poses significant clinical challenges to the effective chemotherapy of many human malignancies. The mechanisms by which cells acquire resistance are multiple and complex, so more extensive understanding of them, as well as identification of novel mechanisms for chemoresistance, will be particularly helpful in providing better therapeutic options. This study is the first report that high levels of ATF4, commonly seen in tumor cells under stressful circumstances, confers gastric cancer cells with a MDR phenotype, and it identifies that this effect is mediated partly by transactivation of SIRT1 expression.

*ATF4* and *SIRT1* are evolutionarily conserved stress response genes involved in a broad spectrum of biological processes, many of which are salutary for homeostasis and cellular protection [22], [23], [24]. Both of these genes are induced in response to a variety of stresses, including oxygen deprivation (hypoxia/anoxia), oxidative stress, DNA damage, nutritional deprivation, and chemotoxic stress. Levenson VV *et al.* first reported that changes in expression of ATF4 could play a role in the pleiotropic resistance to different classes of DNA-targeting drugs [16]. In recent years, several studies had found that ATF4 was involved directly or indirectly in the development of drug resistance through autophagy, the glutathione-dependent redox system, and DNA damage repair [11], [12], [13], [14], [15], [16], [17], [25], [26]. Here we show that the protective ability of ATF4 indeed mediates a MDR phenotype in ATF4-overexpressing gastric cancer cell lines in response to chemotherapy. Our findings clearly show that overexpression of ATF4 in gastric cancer cells was associated with more resistance, while knockdown of ATF4 induced re-sensitization. These data suggest that ATF4 is probably an important downstream mediator of resistance caused by multiple mechanisms and is therefore a valuable therapeutic target. Yet, as one of the most important transcriptional mediators of the ISR which activates a variety of target genes that promote restoration of homeostasis, ATF4 may also mediate resistance by other mechanisms. In our study, SIRT1 was found to be up-regulated in ATF4-overexpressing cells compared to vector transfected cells. In contrast, knockdown of *ATF4* with ATF4 specific siRNA led to a down-regulation of SIRT1 in MDR gastric cancer cells. Our results suggest that SIRT1 might be a downstream mediator of ATF4-induced gastric cancer MDR.

As a member of the ATF subfamily of the basic-region leucine zipper (bZIP) transcription factors [22], ATF4 has the potential to act as either a transcriptional activator or a transcriptional repressor via ATF or cAMP responsive element (CRE) binding sites [22]. The consensus binding site for ATF was defined as TGACGT (C/A) (G/A) [27], which is a sequence identical to the CRE consensus element (TGACGTCA) [28]. Also, the highly conserved core motif – ACGT – in most CREs [29] can bind to different bZIP factors, depending on the flanking bases of the core motif [22], [30], [31]. In our study two putative ATF-CRE binding sites were found in the 1.2 kb *SIRT1* promoter region, and ATF4 directly activated *SIRT1* transcription via binding to both binding elements. However, how the two binding sites play their roles under detailed stress circumstances remains unknown and requires further investigation.

Mammalian *SIRT1* is the closest homologue of the yeast *Sir2* and the most extensively studied SIRT family member. It is heavily implicated in the regulation of cellular processes that determine longevity, including anti-apoptosis, neuronal protection, and cellular senescence or ageing [32]. Recently, an increasing number of studies have implicated increased expression of SIRT1 with resistance to chemotherapy and ionizing radiation [18], [19], [20], [33], [34], [35], [36]. For example, SIRT1 overexpression has been found in drug-resistant neuroblastoma, osteosarcoma, mammary, ovarian, prostate, colon, and lung cancer cell lines compared with their drug-sensitive counterparts. All these drug resistant effects of SIRT1 could possibly be due to its anti-apoptotic effect [37], [38] and silencing of tumor suppressor genes [39]. Finally, we might predict that, if SIRT1 is involved in the ATF4-induced MDR, inhibition of SIRT1 should affect the sensitivity of ATF4-overexpressing cells in response to chemotherapy. As expected, both siRNA and pharmacological inhibition of SIRT1 could re-sensitize ATF4-overexpressing cells to chemical drugs. In addition, our study indicates that SIRT1 protects cells from death partly through an anti-apoptotic effect.

It has been reported that MDR1 was up-regulated in cells with increased SIRT1 expression [18], [36]. In this study, we also demonstrated that MDR1 is up-regulated in ATF4-overexpressing cells, and knockdown of SIRT1 with SIRT1 specific siRNA or inhibiting its activity with EX-527 could lead to down-regulation of MDR1, which is consistent with a drug-resistant role by SIRT1. However, the Bcl-2/Bax ratio, which was up-regulated in the ATF4-overexpressing cells, was SIRT1-independent, suggesting that SIRT1-independent mechanisms also play a role in the ATF4-induced MDR in gastric cancer cells.

In summary, we demonstrate that ATF4 confers a MDR phenotype to gastric cancer cells, and this effect is partly mediated by transactivation of SIRT1 overexpression. Moreover, ATF4 is a valid target in drug-resistant gastric tumors, and developing effective inhibitors of ATF4 should be taken into consideration in the future. These findings provide novel insights into the role of ATF4 in controlling SIRT1 expression and into its stress-resistance features in tumorigenesis and chemotherapy. This is especially important for clinical consideration, as ATF4 can be up-regulated by oxygen deprivation, oxidative stress, nutritional deprivation and almost all the adverse stressors in a tumor microenvironment, which could be hijacked by cancer cells to evade proliferation inhibition and cell death in response to chemotherapy. Therefore, interventions predicated on disrupting stress-induced ATF4 expression in cancer cells may be effective in circumventing or reversing drug resistance in gastric cancer.

## Materials and Methods

For detailed methods, please see Text S1.

### Cell culture and reagents

The human gastric adenocarcinoma cell lines SGC7901 (obtained from the Academy of Military Medical Science, Beijing, China) and the MDR variants, SGC7901/ADR and SGC7901/VCR (established and maintained in our laboratory), and AGS (obtained from the cell bank of Chinese Academy of Sciences, Shanghai, China) were cultured in RPMI-1640 medium supplemented with 10% fetal bovine serum (Hyclone) and penicillin/streptomycin. 293T cells (also obtained from the cell bank of Chinese Academy of Sciences) were cultured in DMEM supplemented with 10% fetal bovine serum. To maintain the MDR phenotype, adriamycin (with a final concentration of 0.5 µg/ml) and vincristine (with a final concentration of 1 µg/ml) were added to the culture media for SGC7901/ADR and SGC7901/VCR cells, respectively. EX-527 (Sigma) was dissolved in DMSO at the indicated concentrations. Adriamycin (ADR), vincristine (VCR), cisplatin (CDDP), and 5-fluorouracil (5-FU) were dissolved in normal saline at indicated concentrations.

### Cell transfection and stable cell lines

The human *ATF4* expression plasmid (pCMV5-ATF4) was kindly provided by Professor Amy S. Lee [25]. Lentiviral vector encoding siRNA specific to *ATF4* and control siRNA were generated with the use of PLKO.1-TRC (Addgene) and were designated as LV-siATF4 and LV-SCR control, respectively. Lentiviral vector encoding human ATF4 gene were constructed in FUW-teto (Addgene), designated as LV-ATF4. The empty vector was used as negative control, designated as LV-Vector. Stable cell lines were generated by transfection of indicated lentiviral constructs followed by selection in puromycin or zeocin (Invitrogen), respectively. Cell transfection and generation of stable cell lines were performed using standard procedures. The sequences of the siRNA constructs can be found in Text S1.

### Immunoblotting

The collection of protein extracts and immunoblotting analysis were performed using standard procedures. For antibody sources, please see Text S1.

### Colony formation assay

The colony formation assay was performed, as previously described [40], with slight modifications (Text S1).

### Annexin V staining and FACS analysis

Annexin V staining and FACS analysis were performed using standard procedures. Cells negative for both PI and Annexin V staining were classified as live cells, cells that stained positive for Annexin V only were classified as early apoptotic cells, and PI positive and Annexin V positive cells were cells undergoing late stages of apoptosis.

### DNA fragmentation assay

DNA fragments were extracted with the DNA Ladder Extraction Kit with Spin Column (C0008, Beyotime Co., Beijing, China) according to the manufacturer's protocol. The DNA fragments were separated using gel electrophoresis on a 1% agarose gel containing 0.1 µg/ml ethidium bromide.

### Hoechst staining

Hoechst Staining was performed according to manufacturer's protocol (C0003, Beyotime Co.). Cells were visualized with a DP70 invert Immunofluorescence microscope (Olympus). Cells with condensed and fragmented nuclei were judged to be apoptotic.

### *In vitro* drug sensitivity assay

ADR, VCR, CDDP, and 5-FU were all freshly prepared before each experiment. Drug sensitivity was measured using a 3-(4,5-dimethylthiazol-2-yl) -2,5-diphenyl-tetrazolium bromide (MTT) assay according to the standard protocol (Text S1).

### Quantitative real-time PCR (qPCR)

Quantitative real-time PCR was performed using a LightCycler 480 II system (Roche) and SYBR Green detection (TaKaRa). Sequences of the primers can be found in Text S1.

### Chromatin immunoprecipitation (ChIP) assay

ChIP assays were performed according to the manufacturer's protocol (P2078, Beyotime Co.) with slight modifications. Chromatin solutions were sonicated and incubated with anti-ATF4 or with control IgG, and rotated overnight at 4°C. DNA–protein cross-links were reversed and chromatin DNA was purified and subjected to PCR analysis. The primers 5′-ACC CCT CGT TTT ACA TCT-3′ and 5′-TTT GGA GTC CTT CCT TTC-3′ were used to amplify the *SIRT1* distal promoter sequence (A1, nucleotides −974 to −843), and the primers 5′-ACC CAA CAA ACC CAT TCT-3′ and 5′-CCT CCT GGG AAG ACC TTT-3′ were used to amplify the *SIRT1* proximal promoter sequence (A2, nucleotides −781 to −647). The primers for *GAPDH*, 5′-TAC TAG CGG TTT TAC GGG CG-3′ and 5′-TCG AAC AGG AGG AGC AGA GAG CGA-3′, were used as a negative control. As a positive control for the ATF4-DNA interaction, the primers 5′-TGG TTG GTC CTC GCA GGC AT-3′ and 5′-CGC TTA TAC CGA CCT GGC TCC T-3′, which were designed to amplify the asparagine synthetase (*ASNS*) promoter region that contains at least two sites reported to bind ATF4 [41], were also used. After amplification, PCR products were resolved on a 1.5% agarose gel and visualized by ethidium bromide staining.

### Reporter gene assay

The 1.2 kb human *SIRT1* promoter sequence (−1100 to +100 bp) was synthesized and cloned into the XhoI and HindIII sites of the pGL3-Basic vector. The resulting construct was confirmed by DNA sequencing. 293T cells were then co-transfected with the *SIRT1* promoter reporter plasmid, the pRL-TK plasmid (Promega, USA), and the pCMV5-ATF4 plasmid by using Lipofectamine2000 (Invitrogen). Forty eight hours after transfection, cells were washed three times with cold phosphate-buffered saline (PBS). Then, the cells were lysed in 100 µl of Passive Lysis Buffer (Promega) and shaken for 15 minutes. Firefly luciferase and Renilla luciferase activities were measured using the Dual-Luciferase Reporter Assay System (Promega) with a Varioskan Flash microplate reader (Thermo Scientific). “Relative activity” was defined as the ratio of firefly luciferase activity to Renilla luciferase activity and was calculated by dividing the luminescence intensity obtained with the assay for firefly luciferase by that of the Renilla luciferase. All measurements were performed in triplicate, and the assays were repeated three times in 293 T cells.

### Statistical analysis

Each experiment was repeated at least three times. All data were presented as mean value ± S.D. The difference between the means was analyzed with Student's t test. All statistical analyses were performed using SPSS16.0 software (Chicago, IL). Significance was set at the 5% level.

## Supporting Information

Figure S1 **Effect of mutated ATF4 binding sites on the activity of the SIRT1 promoter.** 293T cells were co-transfected with pCMV-ATF4 and wild type SIRT1, SIRT1-MUT1, SIRT1-MUT2, or MUT1+MUT2 reporter, and the relative luciferase activity was determined. The luciferase activity of the mock pCMV-Taq group was designated as 1.00. The results are the mean ± S.D. of three experiments performed in duplicate. *, P<0.05. The left side is a schematic representation of the reporter gene constructs. The bar graphs on the right side represent the relative levels of luciferase activity in each of the transfected samples.
(TIF)

Text S1 **Supplementary Material and Methods.**
(DOC)
