# Supplementary figures and images for: Activating Transcription Factor 4 Confers a Multidrug Resistance Phenotype to Gastric Cancer Cells through Transactivation of SIRT1 Expression
Source: PLoS One. 2012 Feb 17;7(2):e31431. doi: 10.1371/journal.pone.0031431 (PMC3281959; doi:10.1371/journal.pone.0031431)

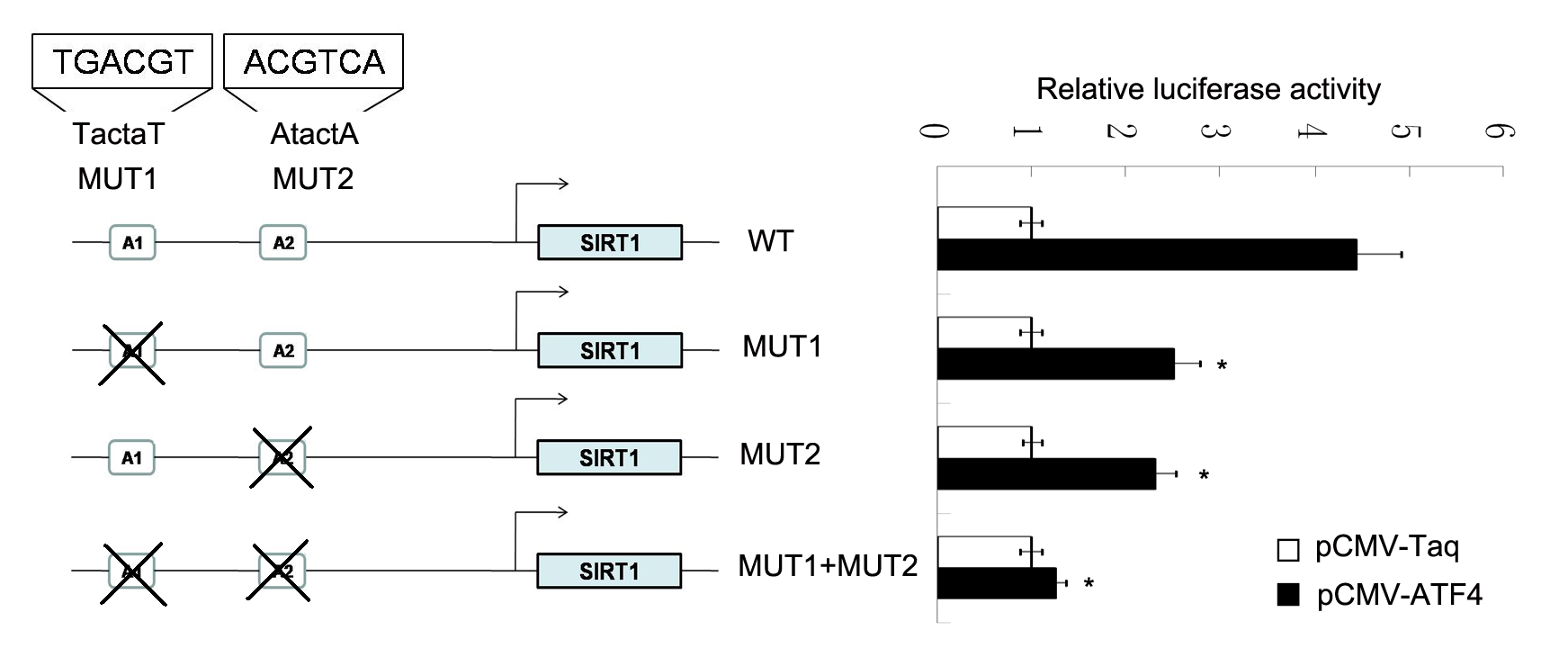

Supplement: Figure S1 — Effect of mutated ATF4 binding sites on the activity of the SIRT1 promoter. 293T cells were co-transfected with pCMV-ATF4 and wild type SIRT1, SIRT1-MUT1, SIRT1-MUT2, or MUT1+MUT2 reporter, and the relative luciferase activity was determined. The luciferase activity of the mock pCMV-Taq group was designated as 1.00. The results are the mean ± S.D. of three experiments performed in duplicate. *, P<0.05. The left side is a schematic representation of the reporter gene constructs. The bar graphs on the right side represent the relative levels of luciferase activity in each of the transfected samples. (TIF) [file pone.0031431.s001.tif]
