# Supplementary material for: Activating Transcription Factor 4 Confers a Multidrug Resistance Phenotype to Gastric Cancer Cells through Transactivation of SIRT1 Expression
Source: PLoS One. 2012 Feb 17;7(2):e31431. doi: 10.1371/journal.pone.0031431 (PMC3281959; doi:10.1371/journal.pone.0031431)
Supplement: Text S1 — Supplementary Material and Methods. (DOC) [file pone.0031431.s002.doc]

**Supplementary Material and Methods**

**Cell transfection and stable cell lines**

The human *ATF4* expression plasmid (pCMV5-ATF4) was kindly provided by Professor Amy S. Lee^1^. Lentiviral vector encoding small interfering RNA (siRNA) specific to *ATF4* (5’-GCA CTT CAA ACC TCA TGG G-3’) and control siRNA (5’-TTC TCC GAA CGT GTC ACG T-3’) were generated with the use of PLKO.1-TRC (Addegene) and were designated as LV-siATF4 and LV-SCR control, respectively. Lentiviral vector encoding human ATF4 gene were constructed in FUW-teto (Addegene), designated as LV-ATF4. The empty vector was used as negative control, designated as LV-Vector. Stable cell lines were generated by transfection of indicated lentiviral constructs followed by selection in puromycin or zeocin (Invitrogen), respectively. Knockdown of *SIRT1* was carried out with either a double-stranded siRNA targeting *SIRT1* (F: 5’-GAA GUU GAC CUC CUC AUU GU dTdT-3’, R: 5’-ACA AUG AGG AGG UCA ACU UC dTdT-3’) described elsewhere^2^ or a scrambled siRNA (F: 5’-UUC UCC GAA CGU GUC ACG U dTdT-3’, R: 5’-ACG UGA CAC GUU CGG AGA A dTdT-3’) using Lipofectamine2000 (Invitrogen).

**Immunoblotting**

Cells were prepared by washing with cold phosphate-buffered saline (PBS) and lysed. Protein concentration was determined using the BSA as standard and Bradford reagent (Bio-Rad). Equal amount of proteins was loaded and separated by SDS–PAGE and transferred onto a 0.22 μm nitrocellulose membrane (Millipore). Blots were probed with anti-ATF4 (sc-200, Santa Cruz), anti-SIRT1 (10E4, Millipore), anti-MDR1 (sc-59593, Santa Cruz), anti-MRP (sc-28289, Santa Cruz), anti-Bcl-2 (sc-7382, Santa Cruz), anti-Bax (sc-70405, Santa Cruz), anti-caspase-3 (sc-271759, Santa Cruz), and anti-β-actin (sc-47778, Santa Cruz). Bound antibodies were visualized using horseradish peroxidase (HRP) conjugated secondary antibodies (Santa Cruz) and enhanced chemiluminescent reagent (Pierce). Quantification was performed using Quantity One software (Bio-Rad).

**Colony formation assay**

The colony formation assay was performed as described elsewhere^3^ with slight modifications. 1×10^3^ cells were seeded into 24-well plates and allowed to adhere for 16 hours. Cells were then treated with CDDP at different concentrations as indicated for 14 days. Then cells were washed with ice-cold phosphate-buffered saline (PBS), fixed with methanol, and stained with crystal violet. The surviving fraction was determined by dividing the number of the surviving colonies in the drug-treated cells by the number of colonies in the non-treated control groups. Quantification was done using Adobe Photoshop as described elsewhere^4^.

**Annexin V staining and FACS analysis**

Cells were trypsinized, washed twice with ice-cold PBS, pH 7.4, and resuspended in 1×binding buffer (10 mM HEPES, pH 7.4, 140 mM NaCl, 2.5 mM CaCl_2_) at a concentration of 1×10^6^ cells/ml. 100 μl of cell suspension was transferred to 4-ml plastic tubes, and 5 μl of Annexin V-fluorescein isothiocyanate (PharMingen) and 4 μl of 0.5 mg/ml PI were added. The cells were gently vortexed and incubated in the dark at room temperature for 15 min. 400 μl of binding buffer was added to each tube, and then subjected to analysis by flow cytometry (FACS). Cells negative for both PI and Annexin V staining were classified as live cells, cells that stained positive for Annexin V only were classified as early apoptotic cells, and PI positive and Annexin V positive cells were cells undergoing late stages of apoptosis.

**DNA fragmentation assay**

For DNA fragmentation assay, after incubation with CDDP at indicated concentrations for 36 hours, cells were collected and washed thrice with PBS. Then the cell pellets were resuspended in PBS and DNA fragments were extracted with the DNA Ladder Extraction Kit with Spin Column (C0008, Beyotime Co., Beijing, China) according to the manufacturer’s protocol. The DNA fragments were separated using gel electrophoresis on a 1% agarose gel containing 0.1 μg /ml ethidium bromide.

**Hoechst staining**

Hoechst Staining was performed according to manufacturer’s protocol (C0003, Beyotime Co.). To examine morphological characteristics of apoptosis, cells were stained with Hoechst 33258 (0.5 ml) at room temperature for 5 min, washed twice in PBS, and visualized with a DP70 invert Immunofluorescence microscope (Olympus). Cells with condensed and fragmented nuclei were judged to be apoptotic.

***In vitro* drug sensitivity assay**

ADR, VCR, CDDP, and 5-FU were all freshly prepared before each experiment. The peak serum concentrations of various anticancer drugs are 2 μg/ml for ADR and VCR, 4 μg/ml for CDDP and 5 μg/ml for 5-FU. Drug sensitivity was measured using a 3-(4,5-dimethylthiazol-2-yl) -2,5-diphenyl-tetrazolium bromide (MTT) assay according to standard protocol. Briefly, cells were trypsinised and diluted with culture medium to the seeding density (5×10^3^ /well), suspended in 96-well flat-bottomed plates (200 μL/well) and incubated at 37℃ for 24 hours. Cells were then incubated in the absence or presence of various concentrations of the anticancer agents. Each condition was assayed in triplicate. After 72 hours, 20 μl of 5 mg/ml MTT (Sigma, St. Louis, Missouri, USA) was added into each well, and cells were cultured for another 4 hours. Then supernatants were discarded and 150 μl of DMSO (Sigma) was added into each well to dissolve crystals. Absorbance at 490 nm was measured with a Varioskan Flash microplate reader (Thermo Scientific, USA). Cell survival fraction were calculated according to formula: survival fraction= (meanA490 of treated wells/meanA490 of untreated wells) × 100%. Finally, dose-effect curves of anticancer drugs were drawn and IC_50_ values were determined.

**Quantitative real-time PCR (qPCR)**

Total RNA was isolated with the Qiagen RNeasy kit (Qiagen), including a DNase I treatment before the final elution to eliminate genomic DNA contamination. cDNA was reverse transcribed from 1.0 mg of total RNA with Random 6 mers and Oligo dT Primer using PrimeScript™ RT reagent Kit (TaKaRa) as recommended by the supplier. Quantitative real-time PCR was performed on equal volumes of cDNA reverse transcription product using a LightCycler 480 II system (Roche) and detection with SYBR Green PCR master mix (TaKaRa). The following primers were used for PCR: glyceraldehyde-3-phosphate dehydrogenase (*GAPDH*), 5’-CGG AGT CAA CGG ATT TGG TCG TAT-3’ and 5’-AGC CTT CTC CAT GGT GGT GAA GAC-3’; *ATF4*, 5’-TCA AAC CTC ATG GGT TCT CC-3’ and 5’-GTG TCA TCC AAC GTG GTC AG-3’; *SIRT1*, 5’-GCT TAT TTG TCA GAG TTC CCA CCC-3’ and 5’-CAG CAT TTT CTC ACT GTT CCA GCC-3’.

**References**

1. Luo S, Baumeister P, Yang S, Abcouwer SF, Lee AS. Induction of Grp78/BiP by translational block: activation of the Grp78 promoter by ATF4 through and upstream ATF/CRE site independent of the endoplasmic reticulum stress elements. J Biol Chem 2003;278:37375-85.

2. Kojima K, Ohhashi R, Fujita Y, Hamada N, Akao Y, Nozawa Y, Deguchi T, Ito M. A role for SIRT1 in cell growth and chemoresistance in prostate cancer PC3 and DU145 cells. Biochem Biophys Res Commun 2008;373:423-8.

3. Carr JR, Park HJ, Wang Z, Kiefer MM, Raychaudhuri P. FoxM1 mediates resistance to herceptin and paclitaxel. Cancer Res 2010;70:5054-63.

4. Lehr HA, Mankoff DA, Corwin D, Santeusanio G, Gown AM. Application of photoshop-based image analysis to quantification of hormone receptor expression in breast cancer. J Histochem Cytochem 1997;45:1559-65.
